# Incremental diagnostic value of 18F-Fluetemetamol PET in differential diagnoses of Alzheimer’s Disease-related neurodegenerative diseases from an unselected memory clinic cohort

**DOI:** 10.1038/s41598-022-14532-z

**Published:** 2022-06-20

**Authors:** Yi-Wen Bao, Yat-Fung Shea, Patrick Ka-Chun Chiu, Joseph S. K. Kwan, Felix Hon-Wai Chan, Henry Ka-Fung Mak

**Affiliations:** 1 Department of Diagnostic Radiology, Li Ka Shing Faculty of Medicine, The University of Hong Kong, Queen Mary Hospital, Room 406, Block K102 Pokfulam Road, Hong Kong SAR, China; 2 Department of Medicine, Queen Mary Hospital, Hong Kong SAR, China

**Keywords:** Cognitive neuroscience, Diagnostic markers

## Abstract

To evaluate the incremental diagnostic value of 18F-Flutemetamol PET following MRI measurements on an unselected prospective cohort collected from a memory clinic. A total of 84 participants was included in this study. A stepwise study design was performed including initial analysis (based on clinical assessments), interim analysis (revision of initial analysis post-MRI) and final analysis (revision of interim analysis post-18F-Flutemetamol PET). At each time of evaluation, every participant was categorized into SCD, MCI or dementia syndromal group and further into AD-related, non-AD related or non-specific type etiological subgroup. Post 18F-Flutemetamol PET, the significant changes were seen in the syndromal MCI group (57%, *p* < 0.001) involving the following etiological subgroups: AD-related MCI (57%, *p* < 0.01) and non-specific MCI (100%, *p* < 0.0001); and syndromal dementia group (61%, *p* < 0.0001) consisting of non-specific dementia subgroup (100%, *p* < 0.0001). In the binary regression model, amyloid status significantly influenced the diagnostic results of interim analysis (*p* < 0.01). 18F-Flutemetamol PET can have incremental value following MRI measurements, particularly reflected in the change of diagnosis of individuals with unclear etiology and AD-related-suspected patients due to the role in complementing AD-related pathological information.

## Introduction

The diagnostic criteria of dementia due to Alzheimer’s Disease (AD) have been updated by incorporating relevant neuroimaging biomarkers, which have greatly improved the distinction of AD at different stages^1–4^. Neuroimaging examination is commonly followed by clinical history, physical examination, cognitive assessments and laboratory tests in a real-life clinical approach^5^. Compared to magnetic resonance imaging (MRI) and other functional imaging modalities such as 18F-Fluorodeoxyglucose positron emission tomography (18F-FDG PET)^6,7^, high cost and radiation exposure of amyloid-PET limits its utility in the real-life clinical setting^8^. Amyloid Imaging Taskforce (AIT) provides Appropriate Use Criteria (AUC) to guide amyloid-PET usage in several specific clinical circumstances only, for instance, patients with persistent or progressively mild cognitive impairment (MCI), possible AD patients presenting with the atypical clinical course or mixed etiologies, and dementia patients with atypically young-onset presentation^9^. However, the AUC has been challenged by previous studies pointing out that the AUC is not able to sufficiently differentiate patients who will and will not benefit from amyloid-PET^10,11^. Correspondingly, the change of etiological diagnosis, increased diagnostic confidence, or altered patient management indicate the diagnostic value of amyloid-PET in MCI and/or dementia reported by previous studies^11–15^.

The use of 18F-Flutemetamol as an amyloid ligand was approved for use by the US Food and Drug Administration (FDA) in 2013. The validation and efficacy of detecting amyloid plaques had been proved by phase I-III studies^16–18^. Although it is in the best interests of dementia specialists for amyloid imaging to be incorporated into daily practice^19^, only a few studies evaluated the clinical value of 18F-Flutemetamol in the tertiary setting^13,15^. In addition, MRI is most likely to be arranged prior to amyloid-PET, but the role of MRI in a change of clinical diagnosis in memory clinics is mentioned less. Through investigation and comparison of two imaging modalities on an unselected memory clinic cohort may give insight to how they provide valuable and complementary information for dementia specialists.

Taken together in the tertiary setting, our study performed a stepwise study design on an unselected cohort following the sequence of clinical evaluation, MRI and 18F-Flutemetamol PET examinations. We aimed to (1) evaluate the effect of MRI on diagnostic change, and (2) investigate whether amyloid-PET with 18F-flutemetemol had incremental value following MRI imaging.

## Methods

### Participants

Cognitively impaired/dementia patients were recruited from the local memory clinic of a university hospital during the period from June 2017 to June 2019. In total, there were 109 participants involved according to the inclusion and exclusion criteria. 15 participants were assessed by 18F-FDG PET test instead of MRI and 10 participants having Pseudo-continuous Arterial spin labeling (PCASL) images with artifacts were excluded. Hence, we had 84 participants included in this study who underwent clinical evaluation, neuropsychological test (local version of Montreal Cognitive Assessment^20^), MRI including structural, MR angiography (MRA), PCASL MR perfusion, and 18F-Flutemetamol PET-CT scanning.

### Inclusion and exclusion criteria

All cognitively impaired or demented patients were required to be aged 55 or over and had an informant such as a caregiver. Any patients with a history of stroke, head injury, seizure, migraine, cancer within the last 5 years, active infection, renal or other organ failures, psychiatric illness, regular alcohol or drug abuse, deafness, or other physical barrier were excluded from the study. In addition, healthy elderlies with prediabetes, diabetes, claustrophobia, and previous cerebrovascular diseases were also excluded. Informed consent was obtained from all non-demented participants, and from the next of kin/caregivers of patients with dementia. Approval of the research protocol by the Institutional Review Board (IRB) of the University of Hong Kong was obtained. The exclusion criteria may be too restrictive in this study. Migraine and diabetes comorbid with cognitive dysfunction are quite common in the general population which may lead to a bias to some degree.

### Data acquisition

#### MRI acquisition

MR images were acquired by a 3 T clinical scanner (Philips Healthcare, Achieva) using a 32-channel head coil at the university imaging center. MRI sequences with parameters as follows: Three-dimensional (3D) T1-weighted MPRAGE using repetition time (TR) = 6.8 ms, echo time (TE) = 3.2 ms, thickness = 1.2 mm, flip angle = 8°, field of view (FOV) = 256 × 240 × 204 (mm), matrix = 256 × 240; 3D FLAIR using TR = 6.8 ms, TE = 3.2 ms, thickness = 1.2 mm, field of view (FOV) = 250 × 250 × 184 (mm), matrix = 208 × 207. 2D Pseudo-continuous ASL with background suppression using single-shot EPI to cover the whole brain with parameters: TR = 4500 ms, flip angle 90°, FOV = 240 × 240 × 119 (mm), matrix = 80 × 77, slices thickness = 7 mm, labeling duration = 1650 ms, post-labeling delay (PLD) = 2000 ms. rs-fMRI with a gradient-echo echo-planar sequence with TR/TE = 2000/30 ms, flip angle = 90$^\circ$, FOV = 230 $\times$ 230 $\times$ 144 (mm), image acquisition resolution = 3.28 $\times$ 3.28 mm^2^, slice thickness = 4 mm, number of volumes = 180. During rs-fMRI, participants were instructed to focus on a cross in the mirror and not to think of anything in particular. In addition, MR angiography of head, susceptibility- and diffusion-weighted images were also acquired. The scanning time of each subject was 45 min in total.

#### 18F-Flutemetamol PET-CT imaging acquisition

All participants were required to fast for at least 6 h and rest in a dimmed room waiting for tracer injection. A bolus of 18F-flutametamol was administered intravenously (within 40 s) to the patients at a dosage of 185Mbq (approximately 5 mCi). The scanning started 90 min after injection, using an integrated in-line PET-CT scanner with 3D list mode. Filtered back-projection reconstruction was used with a slice thickness of 2 to 4 mm, matrix size of 128*128 and the pixel size of 2 mm. A full width half-maximum (FWHM) post-smoothing filter was applied, of 5 mm or less. The duration of the scan was 30 min^16,17^.

### Study design

**Figure 1:**
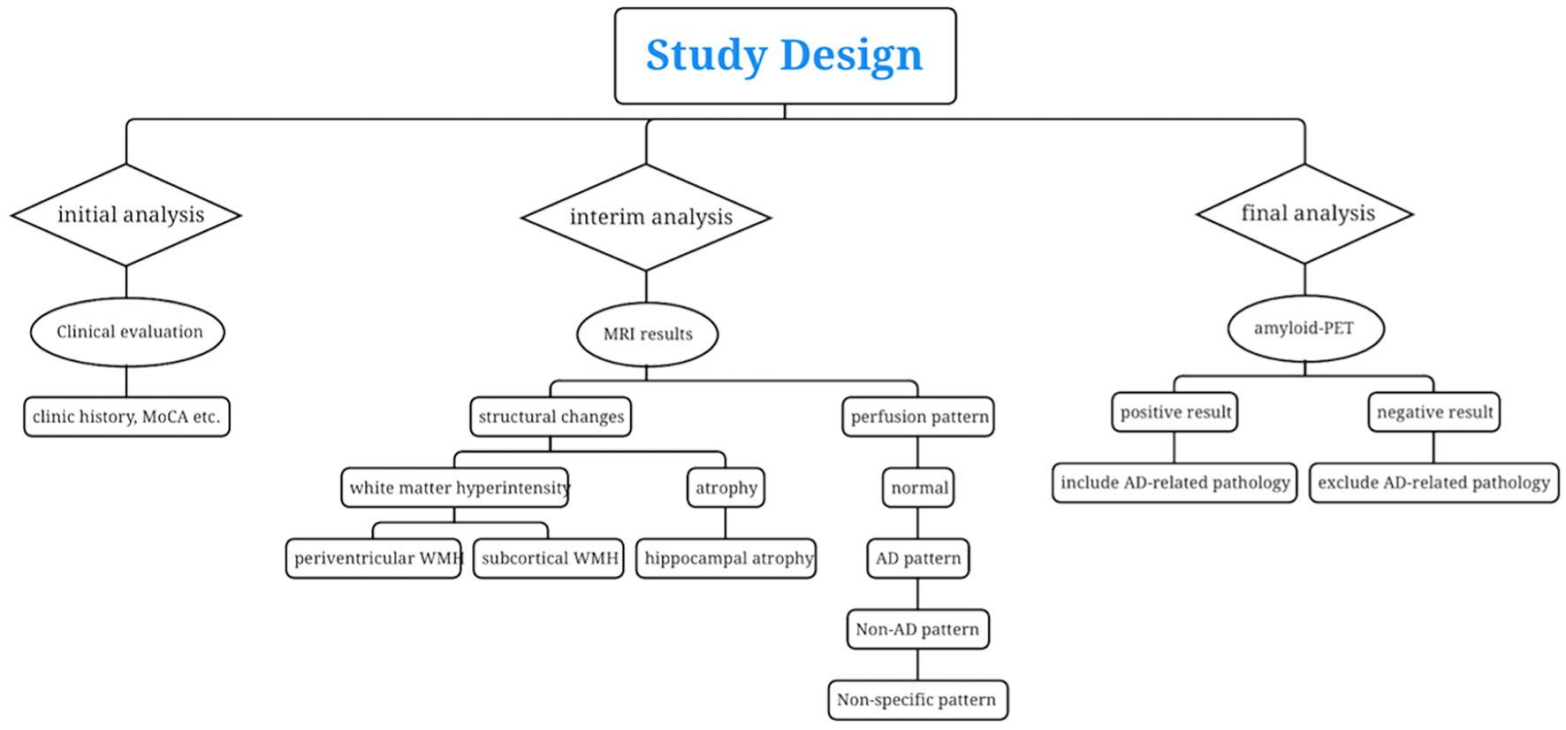
Flowchart of the study and assessments of neuroimaging in cognitive impairment.

The study design consisted of a prospective cohort (n = 84) described above. Three times of diagnoses were made by consensus of a panel consisting of 1 neuroradiologist (HKFM) and 1 geriatrician (YFS). The initial clinical analysis was established by the panel by assessing anonymous clinical information (age, sex, MoCA score, medical history, etc.) of each participant ordered by number without disclosure of MRI and amyloid-PET results. Blinded MRI reports of the same participant were disclosed to our panel for interim analysis to revise the initial diagnosis by assessing the findings of structural MRI, MRI angiography and ASL-MRI. At final analysis, amyloid-PET reports were given for re-evaluation of diagnosis at interim analysis. The final diagnosis of the dementia patients included AD, vascular dementia (VaD), mixed dementia (MD), frontotemporal dementia (FTD), Lewy-body dementia (DLB), and progressive supranuclear palsy (PSP). The panel made the diagnoses of SCD according to Jessen^21^ and MCI according to Peterson^22^. A definitive diagnosis of AD was made based on clinical criteria by McKhann^23^ plus a positive amyloid scan. For VaD, a definitive diagnosis was made based on clinical criteria by Román^24^, plus a negative amyloid scan, microvascular MRI changes or macrovascular MRA abnormalities. The patients fulfilled both AD and VaD were diagnosed as MD. A definitive diagnosis of FTD was according to Neary^25^. Diagnoses of other rarer dementias such as semantic and logopenic variants of primary progressive aphasia according to Montembeault^26^, posterior cortical atrophy according to Crutch^27^, Dementia with Lewy bodies (DLB) according to McKeith^28^, and progressive supranuclear palsy according to Hoglinger^29^. MR perfusion patterns by PCASL could provide supplementary information on a case-by-case basis. At each evaluation, the panel was required to fill in a form with both syndromal and suspected etiological categories for every participant (Table 1). The general flowchart of the study and related assessments of neuroimaging in cognitive impairment is shown in Fig. 1.

**Table 1:** Request form.

Syndromal category	Etiological category
SCD ☐	/
MCI ☐	AD-related ☐
	Non-AD ☐
	Non-specific ☐
DEMENTIA ☐	AD-related ☐
	Non-AD ☐
	Non-specific ☐

### Interpretation of the imaging reports

MRI reports comprise information on periventricular white matter (WM) change, subcortical WM change, hippocampal atrophy, MRA head, and ASL pattern. They were reported by one neuroradiologist (HKFM) at the time 1–2 years before the current study. As shown in Table 2, the grading of periventricular and subcortical WM hyperintensities (WMH) was based on the Fazekas scale^30^, ranging from 0 (normal) to 3 (severe). The extent of atrophy in the hippocampus was graded on the Scheltens scale^31^, ranging from 0 to 4. Both 0 and 1 indicated normal hippocampal structure. The grading of MRA head was dependent on the most severe narrowing of any evaluated intracranial vessel in multiple regions, ordering from normal to severe. For ASL images, they were classified as normal, AD pattern, non-AD pattern, and non-specific pattern, which was consistent with the pattern of metabolism assessed by FDG-PET in suspected dementia^32^. The AD pattern agreed with hypometabolism in the bilateral posterior parietotemporal region and posterior cingulate. The non-AD pattern displayed hypometabolism in other non-AD neurodegenerative disorders or non-typical AD such as Dementia with Lewy body (DLB), Frontotemporal dementia (FTD), and posterior cortical atrophy (PCA). The non-specific pattern was used to label cases with minor patchy focal hypoperfusion.

**Table 2:** MRI and PET variables and the codes used in logistical regression analysis.

Variables	Scale	Coding
Subcortical WM change	Fazekas score	0 (none)
		1 (mild)
		2 (moderate)
		3 (severe)
Periventricular WM change	Fazekas score	0 (none)
		1 (mild)
		2 (moderate)
		3 (severe)
Hippocampal atrophy	Scheltens scale	0/1 (none/minimal)
		2 (mild)
		3 (moderate)
		4 (severe)
MRA head	Normal	0
	Mild	1
	Mild to moderate	2
	Moderate	3
	Moderate to severe	4
	Severe	5
ASL pattern (cat)*	Normal	0
	AD pattern	1
	Non-AD pattern	2
	Non-specific pattern	3
Amyloid	Negative	0
	Positive	1

*ASL pattern was defined as categorical data.

The final 18F-Flutemetamol image of each participant was fused with a raw 18F-Flutemetamol PET image and structural MRI image (3D MPRAGE). Cortex ID (GE Healthcare Ltd., USA), a commercial software, was used in generating and processing the final image including realignment, co-registration, and normalization. The determination of the scan representing positive (abnormal) or negative (normal) was made and reported by a neuroradiologist (HKFM) who had successfully trained for the interpretation of ^18^F-Flutemetamol images through an electronic training program developed by GE Healthcare^33^, at the time 1–2 years before the current study. The specific information about positive or negative image interpretation was listed in the prescribing information document provided by GE Healthcare^34^.

### Syndromal and etiological analysis

As shown in Table 1, the syndromal analysis grouped participants as subjective cognitive decline (SCD)^21^, MCI^22^ or dementia according to each participant’s syndrome. Dementia syndromal groups comprised of patients who fulfilled any clinical criteria of the following syndromes: AD^2^, atypical AD (early-onset AD or AD with posterior cortical atrophy^26^), vascular dementia (VaD)^24^, DLB^28^, FTD^25^, dementia with progressive supranuclear palsy (PSP)^29^, mixed dementia with AD (fulfilled both AD and VD criteria) or non-specific (NS) dementia with the uncertain syndrome.

Etiological analysis was used for the classification of specific subtypes according to each participant’s most likely underlying etiology. Participants were grouped as AD-related, non-AD related, or non-specific types. In subgroups of AD-related types, the underlying etiology of participants was due to AD, including AD, atypical AD, MCI due to AD, and mixed dementia with AD. Non-AD related MCI and non-AD related dementia subgroups were characterized by non-AD etiology such as vascular, FTD, DLB, or PSP components. MCI and dementia patients with unclear etiology were grouped into non-specific (NS) subgroups.

### Statistical analysis

The statistical analysis was performed on SPSS software (SPSS version 23.0.0, Chicago, IL, USA). Our data were non-normally distributed examined by the Shapiro–Wilk test. To compare the differences of continuous variables (MoCA score, age) and ordinal data (Fazekas score of WMH, Scheltens score of hippocampal atrophy, MRA head of stenosis, and amyloid positivity) among groups, the non-parametric Kruskal–Wallis test with multiple comparison corrections was assessed. Categorical variables (sex and ASL pattern) were assessed by the Chi-Square test. The level of significance was set at adjusted *p* < 0.05 and the p values were two-sided. One sample binomial test was applied to assess the proportion of diagnostic change to a significant level after the disclosure of neuroimaging results. The hypothesized proportion was set at 30% (H_0_ = 30%) which was defined as a clinically meaningful threshold of change^35^ and has been applied in previous work^14,36^. Due to the one-sided alternative hypothesis (H_1_ > 30%), the p-value was set at *p* < 0.025. Binary logistic regression analysis was used in assessing the association between MRI findings and diagnostic status (change = 1, not change = 0) at interim analysis, and between amyloid findings and diagnostic status (change = 1, not change = 0) at the final analysis. To perform the analysis, all the MRI and amyloid findings as independent variables were encoded into digits (Table 2). The number (1 to 3) representing different ASL patterns was referenced to the normal ASL pattern (0) since it was a categorical variable. Besides, to control multiple comparisons in the regression model with 5 variables comparing interim analysis to initial analysis, the *p*-value was set at < 0.01. However, only one variable (amyloid positivity) was included in this model comparing the final analysis to interim analysis and the *p*-value remained as 0.05.

### Ethics approval and consent to participate

Informed consent was obtained from all non-demented participants, and from the next of kin/caregivers of patients with dementia. The study logistics complied with the Declaration of Helsinki and ethical approval of the research protocol had been obtained from the Institutional Review Board of the University of Hong Kong.

## Results

### Participants

The demographics and diagnosis of the syndromal groups and etiological subgroups of 84 participants at the initial analysis were presented in Table 3. Most of the participants fell into the syndromal MCI group (41, 49%) and the rest of the participants were clinically diagnosed as dementia (38, 45%) or SCD (5, 6%). In etiological subgroups, AD-related types of MCI (19, 46%) and dementia participants (24, 63%) occupied most in each syndromal group. The MoCA scores were lower in the dementia group than SCD and MCI groups with *p* < 0.0001 and *p* < 0.05. Compared to the SCD group, the MCI group had more severe cognitive impairment with *p* < 0.005.

**Table 3:** Demographics and clinical diagnosis at initial analysis prior to MRI and 18F-Flutemetamol PET imaging.

Demographics based on clinical reading	SCD	MCI	Dementia
No., %	5, 6%	41, 49%	38, 45%
Age, mean (SD)	71.80 (7.89)	76.37 (7.43)	77.84 (7.22)
Sex, % of female	4, 80%	23, 56%	20, 53%
MoCA ^a^, mean (SD)	28.80 (1.30)	20.24 (5.17)	16.53 (6.03)
**Pre-neuroimaging etiological distribution, No., %**			
AD-related	/	19, 46%	24, 63%
Non-AD related type		9, 22%	6, 16%
Non-specific type		13, 32%	8, 21%

^a^MoCA: Dementia < MCI, *p* < 0.05; Dementia < SCD, *p* < 0.0001; MCI < SCD, *p* < 0.05.

### Imaging findings post-MRI

**Figure 2:**
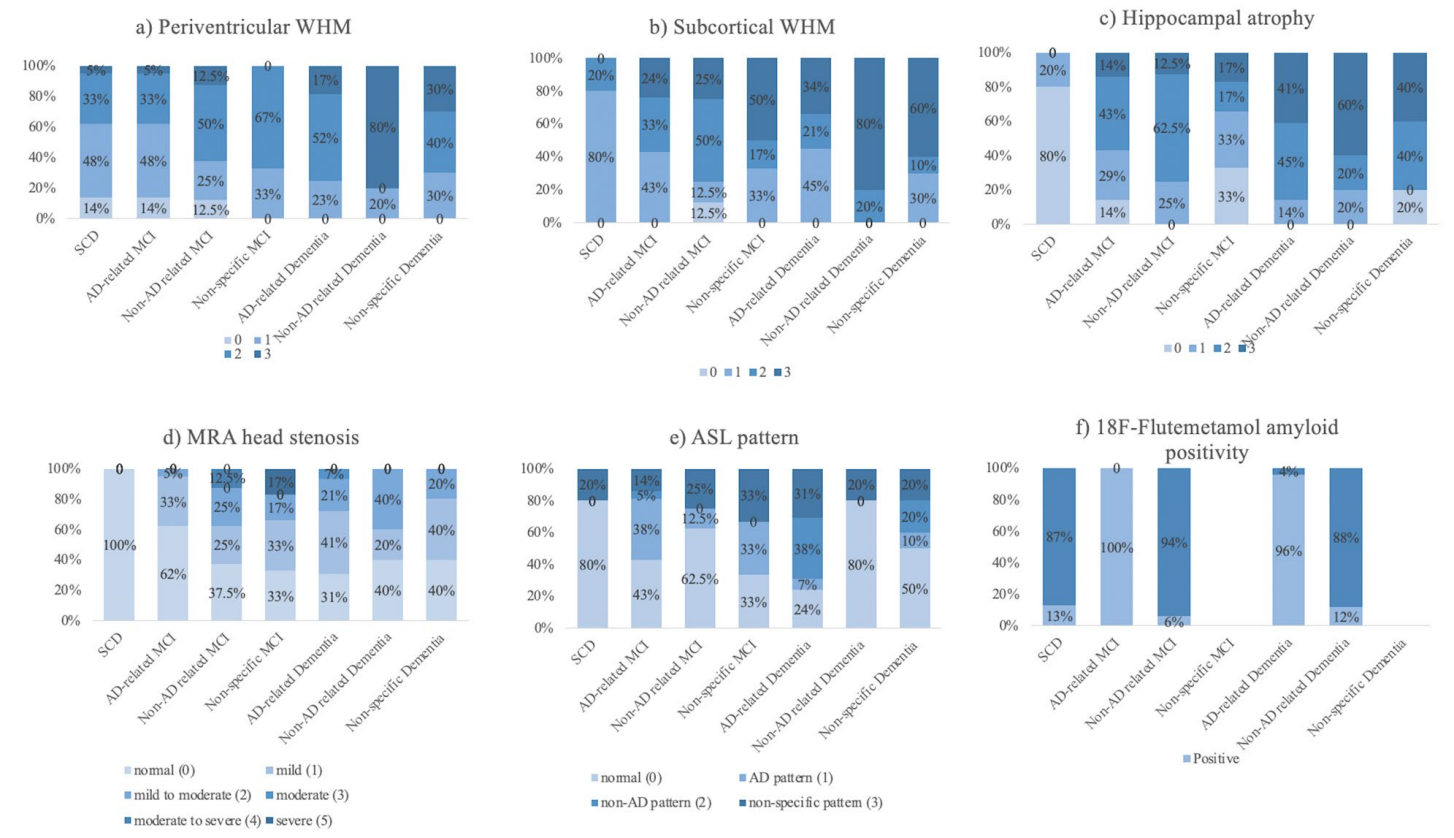
MRI-based findings in each etiological subgroup. (**a**) periventricular WMH; (**b**) subcortical WMH; (**c**) hippocampal atrophy; (**d**) MRA head stenosis; (**e**) ASL pattern and 18F-Flutemetamol-based finding in each etiological subgroup shown as distribution of (**f**) amyloid positivity.

At the interim analysis (Table 4-A), the number of participants in each group was changed to 5 in the SCD group, 35 in the MCI group, and 44 in the dementia group. In Table 4-A, Fig. 2a and b, approximately 62% and 80% of SCD participants showed minimal or mild periventricular and subcortical WM change respectively. All dementia participants had mild or severe WM change (Fazekas score over 1). In the MCI group, the pattern of both periventricular and subcortical WM change was more diffused. Compared to the SCD group (median = 0), the non-AD related dementia subgroup (median = 3) had more server periventricular WM change with *p* < 0.05. Similarly, as shown in Table 4-A and Fig. 2c, 80% of SCD participants were scored on the Scheltens scale with 0/1 illustrating no/minimal (median = 1) hippocampal atrophy. The proportion of participants showing moderate to severe hippocampus atrophy occupied most in all etiological subtypes of dementia (86% in the AD-related dementia subgroup, 80% in the non-AD related dementia subgroup and 80% in the NS dementia subgroup). Compared to the SCD group, AD-related dementia and non-AD related dementia groups had greater atrophy with *p* < 0.01 and *p* < 0.05. The distribution of hippocampal atrophy was also more sporadic in the MCI group. The MRA head stenosis data presented in Table 4-A and Fig. 2d showed no significant difference among all subgroups. ASL pattern mirroring to FDG metabolism suggested that most SCD participants presented with a normal pattern (4, 80%). Interestingly, in the AD-related dementia subgroup, only 2 participants (7%) had a clear AD pattern. The distribution of normal pattern (7, 24%), non-AD pattern (11, 38%), and non-specific pattern (9, 31%) was comparable (Table 4-A, Fig. 2).

**Table 4:** Diagnostic results and post-neuroimaging findings.

Diagnostic Analysis	Variables	SCD (*n* = 5, 6%)	MCI (*n* = 35, 42%)			Dementia (*n* = 44, 52%)		
	Subtype No., % of each syndromal group	/	AD-related	Non-AD related	Non-specific	AD-related	Non-AD related	Non-specific
		5, 100%	21, 60%	8. 23%	6, 18%	29, 66%	5, 11%	10, 22%
**(A)**								
At interim analysis	**MRI findings**							
	Periventricular Fazekas score ^**a**^, No							
	0	3	3	1	0	0	0	0
	1	1	10	2	2	9	1	3
	2	1	7	4	4	15	0	4
	3	0	1	1	0	5	4	3
	**Median**	**0**	**1**	**2**	**2**	**2**	**3**	**2**
	Subcortical Fazekas score, No							
	0	0	0	1	0	0	0	0
	1	4	9	1	2	13	0	3
	2	1	7	4	1	6	1	1
	3	0	5	2	3	10	4	6
	**Median**	**1**	**2**	**2**	**2.5**	**2**	**3**	**3**
	Scheltens Scale ^**b**^, No							
	0/1	4	3	0	2	0	0	2
	2	1	6	2	2	4	1	0
	3	0	9	5	1	13	1	4
	4	0	3	1	1	12	3	4
	**Median**	**1**	**3**	**3**	**2**	**3**	**4**	**3**
	MRA head, No							
	Normal (0)	5	13	3	2	9	2	4
	Mild (1)	0	7	2	2	12	1	4
	Mild to moderate (2)	0	1	2	1	6	2	2
	Moderate (3)	0	0	0	0	2	0	0
	Moderate to severe (4)	0	0	1	0	0	0	0
	Severe (5)	0	0	0	1	0	0	0
	**Median**	**0**	**0**	**1**	**1**	**1**	**1**	**1**
	ASL pattern, No							
	Normal (0)	4	9	5	2	7	4	5
	AD pattern (1)	0	8	1	2	2	0	1
	Non-AD pattern (2)	0	1	0	0	11	0	2
	Non-specific pattern (3)	1	3	2	2	9	1	2

Diagnostic analysis	Variables	SCD (*n* = 8, 10%)	MCI (*n* = 32, 38%)			Dementia (*n* = 44, 52%)		
	Subtype No., % of each syndromal group	/	AD-related	Non-AD related	Non-specific	AD-related	Non-AD related	Non-specific
		8, 100%	11, 34%	21, 66%	0	27, 61%	17, 39%	0
**(B)**								
At final analysis	**18F-Flutemetamol PET findings**							
	Positivity of amyloid scanning ^c^, No	1	11	2	0	26	2	0

(A) post-MRI, (B) post-18F-Flutemetamol PET.

^a^periventricular Fazekas score: SCD vs. non-AD related dementia, *p* < 0.05.

^b^Scheltens scale: SCD vs. AD-related dementia, *p* < 0.01; SCD vs non-AD related dementia, *p* < 0.05.

^c^amyloid positivity: SCD vs. AD-related dementia, *p* < 0.0001; SCD vs AD-related MCI, *p* < 0.01; AD-related MCI vs. non-AD related MCI, *p* < 0.0001; AD-related MCI vs non-AD related dementia, *p* < 0.0001; non-AD related MCI vs. AD-related dementia, *p* < 0.0001; non-AD related dementia vs. AD-related dementia, *p* < 0.0001.

### Changes in diagnostic results post-MRI

Post MRI, 18 MCI (44%) and 11 dementia participants (29%) had a new diagnosis (Table 5-A). In Table 5-B, the proportional changes were predominant found in subgroups of NS MCI, non-AD related MCI, and non-AD related dementia, displaying 67%, 69%, and 67% respectively. The change in non-AD related MCI (67%) had significant results of *p* < 0.025 due to 6 of them being recategorized to 3 AD-related dementia, 2 NS MCI, and 1 NS dementia, compared to the clinically meaningful threshold of diagnostic change (30%). In NS MCI participants, 2 of them were revised to MCI with AD-related etiology and 7 of them were revised as MCI/dementia with non-AD related etiology leading to a significant result with *p* < 0.01 compared to the threshold (30%).

**Table 5:** Change in diagnosis following MRI.

Syndromal diagnosis at initial analysis	Change post-MRI in syndromal group, No., %	Comparing to priori threshold (30%), *p* value	
**(A)**			
SCD (*n* = 5)	0	/	
MCI (*n* = 41)	18, 44%	*p* > 0.025	
Dementia (*n* = 38)	11, 29%	*p* > 0.025	
Total (*n* = 84)	29, 35%	*p* > 0.025	

Etiological diagnosis at initial analysis	Change post-MRI in etiological subgroup, No., %	Comparing to priori threshold (30%), *p* value	Diagnosis post-MRI of changed subjects
**(B)**			
SCD (*n* = 5)	0	/	/
AD-related MCI (*n* = 19)	3, 16%	*p* > 0.025	2 AD-related Dementia, 1 Non-AD related MCI
Non-AD related MCI (*n* = 9)	6, 67%	***p* < 0.025**	3 AD-related Dementia, 2 NS MCI, 1 NS Dementia
Non-specific MCI (*n* = 13)	9, 69%	***p* < 0.01**	2 AD-related MCI, 5 Non-AD MCI, 2 Non-AD related Dementia
AD-related Dementia (*n* = 24)	4, 17%	*p* > 0.025	3 NS dementia, 1 Non-AD related Dementia
Non-AD related Dementia (*n* = 6)	4, 67%	*p* > 0.025	3 AD-related Dementia, 1 NS Dementia
Non-specific Dementia (*n* = 8)	3, 38%	*p* > 0.025	2 AD-related Dementia, 1 Non-AD related Dementia

Significant values are in [bold].

(A) in the syndromal group, (B) in the etiological subgroup.

*NS* Non-specific.

### Imaging findings post-18F-Flutemetamol PET-CT

Post 18F-Flutemetamol PET-CT, the overall distribution in syndromal groups was 10% in the SCD group involving 8 participants, 38% in the syndromal MCI group including 32 participants, and 52% in the syndromal dementia group consisting of 44 participants (Table 4-B). At final analysis, all NS MCI or NS dementia participants were diagnosed with specific etiologies. All AD-related MCI and 96% of AD-related dementia participants had positive amyloid scanning. In contrast, 94% of non-AD related MCI and 88% of non-AD dementia participants showed negative amyloid results. Besides, 13% of SCD participants had positive 18F-flutemetamol-PET scans (Table 4-B, Fig. 2f). The significant differences in amyloid positivity were seen when comparing SCD vs AD-related dementia (*p* < 0.0001), SCD vs AD-related MCI (*p* < 0.01), AD-related MCI vs non-AD related MCI (*p* < 0.0001), AD-related MCI vs non-AD related dementia (*p* < 0.0001), non-AD related MCI vs AD-related dementia (*p* < 0.0001) and non-AD related dementia vs AD-related dementia (*p* < 0.0001).

### Changes in diagnostic results post-18F-Flutemetamol PET-CT

The overall diagnostic change was up to 56% (*p* < 0.0001) post 18F-Flutemetamol PET-CT in Table 6-A. Compared to the 30% threshold, significant changes were seen in the syndromal MCI group (57%, *p* < 0.001) involving the following etiological subgroups: AD-related MCI (57%, *p* < 0.01), NS MCI (100%, *p* < 0.0001); and syndromal dementia group (61%, *p* < 0.0001) consisting of NS dementia subgroup (100%, *p* < 0.0001) shown in Table 6-A and -B. In addition, 48% of AD-related dementia participants were re-evaluated to MCI or dementia with non-AD related etiology due to negative amyloid scans. 9 out of 21 AD-related MCI participants were changed to the non-AD related MCI subgroup due to the negative amyloid results.

**Table 6:** Change in diagnosis following 18F-Flutemetamol PET.

Syndromal diagnosis at interim analysis	Change post-18F-Flutemetamol PET in syndromal group, No. (%)	Comparing to priori threshold (30%), *p* value			
**(A)**					
SCD (*n* = 5)	0	/			
MCI (*n* = 35)	20, 57%	***p* < 0.001**			
Dementia (*n* = 44)	27, 61%	***p* < 0.0001**			
Total	47, 56%	***p* < 0.0001**			

Etiological diagnosis at interim analysis	Change post-18F-Flutemetamol PET findings in etiological subgroup, No., %	Comparing to priori threshold (30%), *p* value	Amyloid status post-18F-Flutemetamol PET	No. of subjects in each amyloid status	Diagnosis post-18F-Flutemetamol PET of changed subjects
**(B)**					
SCD (*n* = 5)	0	/	Positive	/	/
			Negative		
AD-related MCI (*n* = 21)	12, 57%	***p* < 0.01**	Positive	3	1 SCD, 2 AD-related Dementia, 9 Non-AD related MCI
			Negative	9	
Non-AD related MCI (*n* = 8)	2, 25%	*p* > 0.025	Positive	1	1 AD-related MCI, 1 Non-AD related dementia
			Negative	1	
Non-specific MCI (*n* = 6)	6, 100%	***p* < 0.0001**	Positive	3	1 SCD, 3 Non-AD related MCI, 2 AD-related Dementia
			Negative	3	
AD-related Dementia (*n* = 29)	14, 48%	*p* > 0.025	Positive	0	3 Non-AD related MCI, 11 Non-AD related Dementia
			Negative	14	
Non-AD related Dementia (*n* = 5)	3, 60%	*p* > 0.025	Positive	1	2 AD-related Dementia, 1 Non-AD related MCI
			Negative	2	
Non-specific Dementia (*n* = 10)	10, 100%	***p* < 0.0001**	Positive	8	1 SCD, 3 Non-AD related MCI, 6 AD-related Dementia
			Negative	2	

(A) in the syndromal group, (B) in the etiological subgroup. Significant values are in bold.

### Changes in diagnostic results post-MRI and 18F-Flutemetamol PET-CT

The overall diagnostic change was up to 65% (*p* < 0.0001) post-MRI and 18F-Flutemetamol PET-CT shown in Table 7-A. Syndromal MCI and dementia groups illustrated 78% and 61% with *p* < 0.0001 respectively. In Table 7-B, the proportional changes were predominant in most of the etiological subgroups compared to the 30% threshold including AD-related MCI (63%, *p* < 0.01), non-AD related MCI (78%, *p* < 0.01), NS MCI (100%, *p* < 0.0001), AD-related dementia (54%, *p* < 0.025) and NS dementia subgroups (100%, *p* < 0.0001). In addition, 9 out of 12 AD-related MCI individuals and 12 out of 13 AD-related dementia individuals at initial analysis were changed to non-AD related MCI/dementia group with negative amyloid scans.

**Table 7:** Change in diagnosis following MRI and 18F-Flutemetamol PET.

Syndromal diagnosis at interim analysis	Change post-MRI and 18F-Flutemetamol PET in syndromal group, No. (%)	Comparing to priori threshold (30%), *p* value			
**(A)**					
SCD (*n* = 5)	0	/			
MCI (*n* = 35)	32, 78%	***p* < 0.0001**			
Dementia (*n* = 44)	23, 61%	***p* < 0.0001**			
Total	55, 65%	***p* < 0.0001**			

Etiological diagnosis at interim analysis	Change post-MRI and 18F-Flutemetamol PET findings in etiological subgroup, No., %	Comparing to priori threshold (30%), p value	Amyloid status post-neuroimaging	No. of subjects in each amyloid status	Diagnosis post-neuroimaging of changed subjects
**(B)**					
SCD (*n* = 5)	0	/	Positive	/	/
			Negative		
AD-related MCI (*n* = 19)	12, 63%	***p* < 0.01**	Positive	3	7 Non-AD related MCI, 3 AD-related Dementia, 2 Non-AD related Dementia
			Negative	9	
Non-AD related MCI (*n* = 9)	7, 78%	***p* < 0.01**	Positive	5	2 SCD, 1 AD-related MCI, 1 Non-AD related Dementia, 3 AD-related Dementia
			Negative	2	
Non-specific MCI (*n* = 13)	13, 100%	***p* < 0.0001**	Positive	7	2 AD-related MCI, 8 Non-AD related MCI, 3 AD-related Dementia
			Negative	6	
AD-related Dementia (*n* = 24)	13, 54%	***p* < 0.025**	Positive	1	1 AD-related MCI, 4 Non-AD related MCI, 8 Non-AD related Dementia
			Negative	12	
Non-AD related Dementia (*n* = 6)	2, 23%	p > 0.025	Positive	1	2 AD-related Dementia
			Negative	1	
Non-specific Dementia (*n* = 8)	8, 100%	***p* < 0.0001**	Positive	6	1 SCD, 6 AD-related Dementia, 2 Non-AD related Dementia
			Negative	2	

Significant values are in [bold].

(A) in the syndromal group, (B) in the etiological subgroup.

### Multivariable analysis of factors associated with diagnostic change post-MRI and post-18F-Flutemetamol PET

In the binary logistic regression model (Table 8), comparing the interim analysis to the initial analysis, the effects of 5 MRI variables on diagnostic change showed no significant contribution. However, amyloid status had a significant influence on the diagnostic results of interim analysis (*p* < 0.01), with an odds ratio (OR) of 3.333 (95%CI: 1.347–8.252).

**Table 8:** Binary logistic regression in the association between neuroimaging variables and diagnostic change.

Diagnostic analysis	Variables	*p*-value	B^a^	Exp (B) ^b^ (95% CI)
Interim analysis	periventricular WM change	0.288	0.524	1.689 (0.643–4.441)
	subcortical WM change	0.073	− 0.815	0.443 (0.182–1.079)
	hippocampal atrophy	0.565	− 0.158	0.854 (0.499–1.079)
	MRA head	0.176	0.354	1.425 (0.853–2.382)
	ASL pattern (0)	0.451	/	/
	ASL pattern (1)	0.232	0.797	2.218 (0.601–8.184)
	ASL pattern (2)	0.907	− 0.1	0.906 (0.167–4.901)
	ASL pattern (3)	0.297	0.883	2.481 (0.461–12.682)
Final analysis	positive amyloid result	** < 0.01**	1.204	3.333 (1.347–8.252)

Significant values are in [bold].

^a^B is logistic regression coefficient.

^b^Exp (B), or the odds ratio, refers to the exponential value of B illustrating the predicted change in odds for a unit increase in the predictor.

## Discussion

The role of MRI and amyloid-PET has been widely validated and employed in aiding with the clinical diagnosis of dementia by investigating the underlying etiology, particularly for AD, in recent years. Our stepwise study design, including an unselected population from a tertiary memory clinic, encompassing SCD, MCI, and dementia participants, illustrated that both MRI and amyloid-PET with 18F-Flutemetamol resulted in different etiological subgroups at a statistical level. In addition, following MRI measurements, 18F-Flutemetamol PET can have incremental value by complementing more specific AD-related pathological information, resulting in a change of diagnosis.

At the interim analysis, the periventricular WMH and hippocampal atrophy assessed by structural MRI were well-differentiated between the SCD and dementia subgroups, which were accordant with previous work^37–40^. However, it is surprising to find that there was no significant difference in vascular and structural changes between our AD-related MCI/dementia subgroups and non-AD related MCI/dementia subgroups. One possible cause could be the MCI and dementia participants with mixed etiologies were categorized in our AD-related subgroups. Mixed pathologies were commonly observed in the elderly with MCI^41^ or dementia^42^. According to one study, approximately 45.8% of pathologically-confirmed AD subjects had mixed pathologies, especially with macroscopic infarcts occupying approximately 67%. 19.4% of MCI patients had mixed pathologies which was also reported^43^. Therefore, the sole use of MRI in the diagnosis of MCI/dementia with mixed etiologies may not be satisfactory. Besides, PCASL MR perfusion can detect cerebral blood flow efficiently and show specific hypoperfusion patterns in AD markedly distinct from other types of dementia^44,45^ and MCI^46,47^. However, the significant distinction was not shown in our results, which may be due to our limited sample size. The 2D EPI PCASL technique used in our study observed worse repeatability and less reliability for imaging than 3D spin echo PCASL^48^.

Most of the diagnostic change due to MRI results was reflected in the syndromal MCI groups and half of these patients initially diagnosed with MCI were changed to dementia. As explained by Jack et al.^49^, participants with reserved cognition may have advanced evidence of biomarkers due to the subject-specific lag in time between cognitive impairment and the presence of biomarkers. Nevertheless, the number of patients with unclear etiology (6 NS MCI and 10 NS dementia patients) after incorporating MRI findings, which was comparable with that (13 NS MCI and 8 NS dementia) at initial analysis. Additionally, only a small proportion of AD-like hypoperfusion was shown in AD-related dementia in our result. Although structural atrophy or regional hypometabolism measured by structural MRI or FDG-PET (similar to ASL perfusion) which were commonly considered as a biomarker closely associated with symptom severity throughout the AD spectrum, they could only provide less specific information about neurodegeneration and neural injuries that could be found in lots of disorders^50–52^.

At the final analysis, the overall diagnostic change was 56% for all participants (*p* < 0.001) in our study, which fell in a range of 9–68.8% based on a systematic review^53^. The high proportion of changes in diagnosis was mainly due to the change from NS MCI and NS dementia to a confirmed etiological subgroup. This finding was also consistent with the Leuzy et al. study showing that the highest percentage of diagnostic change was in those with dementia not otherwise specified^13^. Aβ as a more specific pathological biomarker in AD^54^ commonly was believed to be the dominant factor in excluding AD pathology^9^, such as excluding AD as a cause of Aβ disorders^55^. As shown in this study, 9 out of 12 AD-related MCI and 14 out of 14 AD-related dementia patients diagnosed at interim reading showed negative amyloid results and thus changed to non-AD related MCI/dementia. Compared to the change at interim analysis mainly reflected in the syndromal MCI group, the 18F-Flutemetamol PET findings influenced the diagnoses of both syndromal MCI and dementia groups (61% of dementia patients changed post 18F-Flutemetamol PET with *p* < 0.0001). MCI patients have a high likelihood of progressing to clinical AD, especially individuals with positive amyloid deposition^56–59^. Although the magnitude of neurodegeneration assessed by MRI is closely coupled with cognitive decline, this biomarker is seen in lots of disorders^49^. In Vemuri et al. study^60^, MRI and CSF assessing brain atrophy, and total tau and Aβ_1-42_ respectively, provided complementary information for predicting amnesic MCI into AD. Additionally, the combination of the biomarkers showed better prediction than either source alone. The amyloid imaging may take an important role in determining which MCI individuals are likely to benefit from early intervention or therapies. This finding gives us a hint for enhancing the clinical diagnoses of MCI/AD or even predicting the conversion of MCI to AD by a combination of amyloid-PET and MRI modalities. The incremental diagnostic value of 18F-Flutemetamol PET following MRI was also reflected in our logistic regression analysis. Amyloid status had an essential effect on change in diagnosis (*p* < 0.01). Supported by the Rabinovici et al. study^14^, the amyloid-PET result also had a significant association with change in the composite management with *p* < 0.001.

SCD as a high-risk population of AD are associated with elevated amyloid deposition^61^ and may further progress into AD after follow-up^62,63^. In this study, the 5 participants initially diagnosed as SCD based on clinical evaluation remained in the same diagnostic state despite post-MRI or post-PET reading. Compared to previous studies reporting 60% of SCD subjects had changed diagnostic results^13^, and 23% of the cases interchanged between AD suspected etiology and non-AD suspected etiology post-amyloid-PET imaging^11^, our result could be due to the low prevalence of amyloid positivity in our SCD group (1 out of 8, 13%) and 5 of them remained the same diagnosis due to negative amyloid results.

The diagnostic change at the final analysis involved most of the etiological subgroups at the statistical level except for the non-AD related dementia subgroup compared to the initial analysis. The small size of the non-AD dementia subgroup (*n* = 6) at the initial diagnosis may lead to the result. It is not surprising to see the finding since MRI and amyloid-PET can reflect the major cerebral changes in lots of neurodegenerative diseases, particularly AD. When comparing the change post-MRI with that post-MRI and 18F-Flutemetamol PET, AD-related MCI/dementia subgroups showed additional significant results as individuals changed non-AD related MCI/dementia category showing negative PET scans. Although the diagnostic change post-18F Flutemetamol PET alone was not assessed in this study, previous studies have proved that a combination of these neuropathological factors assessed by multiple modalities improves the likelihood of AD etiology resulting in the improvement of clinical diagnosis, even at MCI stage^47,64–66^.

### Limitations

One of the limitations of our study was the small sample size. Furthermore, the threshold of 30% applied in our study may not have similar statistical power as the previous study due to the limited sample size. The exclusion criteria may be too restrictive in this study, such as migraine and diabetes comorbid with cognitive dysfunction, which are quite common in the general population. Our study is a retrospective study and is not able to assess the changes in the management plan of patients and pre-or post-imaging confidence of diagnosis from our specialist. Besides, both MRI and 18F-Flutemetamol PET imaging results were assessed by one neuroradiologist 1–2 years before the current study. The interpretations of images could be largely dependent on personal training and experience.

## Conclusion

In summary, our study reports that both MRI and amyloid imaging can lead to a change of diagnosis. MRI as a first-line investigation of neuroimaging in clinical assessments provides efficient information about neurodegeneration but is less specific for AD-related pathology. Meanwhile, amyloid-PET with 18F-Flutemetamol can have incremental value following MRI measurements, particularly reflected in the change of diagnosis of individuals with unclear etiology and AD-related-suspected patients due to the role in complementing AD-related pathological information.

## Data Availability

The datasets generated and/or analyzed during the current study are not publicly available for patient privacy protection purposes but are available from the corresponding author on reasonable request.

## Abbreviations

SCD: Subjective cognitive decline
MCI: Mild cognitive impairment
AD: Alzheimer’s disease
AIT: Amyloid imaging taskforce
AUC: Appropriate use criteria
ASL: Arterial spin labeling
DLB: Dementia with Lewy body
FTD: Frontotemporal dementia
MRI: Magnetic resonance imaging
NS: Non-specific
PCA: Posterior cortical atrophy
PCASL: Pseudo-continuous arterial spin labeling
PET: Positron emission tomography
PSP: Progressive supranuclear palsy
VaD: Vascular dementia
WM: Whit matter
WMH: Whit matter hyperintensity
18F-FDG PET: 18F-Fluorodeoxyglucose positron emission tomography

## References

[1] Chételat G (2018). Multimodal neuroimaging in Alzheimer's disease: early diagnosis, physiopathological mechanisms, and impact of lifestyle. J. Alzheimers Dis..

[2] McKhann GM (2011). The diagnosis of dementia due to Alzheimer's disease: Recommendations from the National Institute on Aging-Alzheimer's Association workgroups on diagnostic guidelines for Alzheimer's disease. Alzheimers Dement..

[3] Sperling RA (2011). Toward defining the preclinical stages of Alzheimer's disease: recommendations from the National Institute on Aging-Alzheimer's Association workgroups on diagnostic guidelines for Alzheimer's disease. Alzheimers Dement.

[4] Dubois B (2016). Preclinical Alzheimer's disease: Definition, natural history, and diagnostic criteria. Alzheimers Dement.

[5] Lam K (2019). Assessment and diagnosis of dementia: a review for primary healthcare professionals. Hong Kong Med. J..

[6] England, N., *2014/15 National Tariff Payment System*. (NHS England Publications, 2013).

[7] Rayment D (2016). Neuroimaging in dementia: an update for the general clinician: Neuroimaging in dementia. Prog. Neurol. Psychiatr. (Guildf.).

[8] Royal College of Physicians of Edinburgh, and Administration of Radioactive Substances Advisory Committee. Evidence-based indications for the use of PET-CT in the United Kingdom 2016. Clin. Radiol. **71**(7), e171–88 (2016). 10.1016/j.crad.2016.05.001 27207376

[9] Johnson KA (2013). Appropriate use criteria for amyloid PET: A report of the Amyloid Imaging Task Force, the Society of Nuclear Medicine and Molecular Imaging, and the Alzheimer's Association. Alzheimer's Dement..

[10] de Wilde A (2019). Assessment of the appropriate use criteria for amyloid PET in an unselected memory clinic cohort: The ABIDE project. Alzheimer's Dement..

[11] de Wilde A (2018). Association of amyloid positron emission tomography with changes in diagnosis and patient treatment in an unselected memory clinic Cohort: The ABIDE project. JAMA Neurol..

[12] Grundman M (2013). Potential impact of amyloid imaging on diagnosis and intended management in patients with progressive cognitive decline. Alzheimer Dis. Assoc. Disord..

[13] Leuzy A (2019). Clinical impact of [18 F]flutemetamol PET among memory clinic patients with an unclear diagnosis. Eur. J. Nucl. Med. Mol. Imaging.

[14] Rabinovici GD (2019). Association of amyloid positron emission tomography with subsequent change in clinical management among medicare beneficiaries with mild cognitive impairment or dementia. JAMA.

[15] Zwan MD (2017). Diagnostic impact of [(18)F]flutemetamol PET in early-onset dementia. Alzheimer's Res. Ther..

[16] Nelissen N (2009). Phase 1 study of the Pittsburgh compound B Derivative ^sup 18^F-Flutemetamol in healthy volunteers and patients with probable Alzheimer disease. J. Nucl. Med..

[17] Vandenberghe R (2010). 18F-flutemetamol amyloid imaging in Alzheimer disease and mild cognitive impairment: A phase 2 trial. Ann. Neurol..

[18] Curtis C (2015). Phase 3 trial of flutemetamol labeled with radioactive fluorine 18 imaging and neuritic plaque density. JAMA Neurol..

[19] Klein EP, Kaye J (2013). Dementia specialists and early adoption of amyloid imaging. J. Alzheimers Dis..

[20] Wong A (2009). The validity, reliability and clinical utility of the hong kong montreal cognitive assessment (HK-MoCA) in patients with cerebral small vessel disease. Dement. Geriatr. Cogn. Disord..

[21] Jessen F (2014). A conceptual framework for research on subjective cognitive decline in preclinical Alzheimer's disease. Alzheimer's Dement..

[22] Petersen RC (1999). Mild cognitive impairment: clinical characterization and outcome. Arch. Neurol..

[23] McKhann GM (2011). The diagnosis of dementia due to Alzheimer’s disease: recommendations from the National Institute on Aging-Alzheimer’s Association workgroups on diagnostic guidelines for Alzheimer's disease. Alzheimer's Dement..

[24] Román GC (1993). Vascular dementia: diagnostic criteria for research studies Report of the NINDS-AIREN International Workshop. Neurology.

[25] Neary D (1998). Frontotemporal lobar degeneration: a consensus on clinical diagnostic criteria. Neurology.

[26] Montembeault M (2018). Clinical, anatomical, and pathological features in the three variants of primary progressive aphasia: a review. Front. Neurol..

[27] Crutch SJ (2017). Consensus classification of posterior cortical atrophy. Alzheimer's Dement..

[28] McKeith IG (2017). Diagnosis and management of dementia with Lewy bodies: Fourth consensus report of the DLB Consortium. Neurology.

[29] Höglinger GU (2017). Clinical diagnosis of progressive supranuclear palsy: The movement disorder society criteria. Mov. Disord..

[30] Fazekas F (1987). MR signal abnormalities at 1.5 T in Alzheimer's dementia and normal aging. AJR Am. J. Roentgenol..

[31] Scheltens P (1992). Atrophy of medial temporal lobes on MRI in "probable" Alzheimer's disease and normal ageing: diagnostic value and neuropsychological correlates. J. Neurol. Neurosurg. Psychiatry.

[32] Brown RK (2014). Brain PET in suspected dementia: patterns of altered FDG metabolism. Radiographics.

[33] Buckley JC (2017). Validation of an electronic image reader training programme for interpretation of [18F]flutemetamol β-amyloid PET brain images. Nucl. Med. Commun..

[34] *Flutemetamol F 18 Injection*. 2020, GE Healthcare.

[35] Hillner BE (2007). The national oncologic PET registry (NOPR): design and analysis plan. J. Nucl. Med..

[36] Grundman M (2013). Potential impact of amyloid imaging on diagnosis and intended management in patients with progressive cognitive decline. Alzheimer Dis. Assoc. Dis..

[37] Duara R (2008). Medial temporal lobe atrophy on MRI scans and the diagnosis of Alzheimer disease. Neurology.

[38] Clerx L (2013). Measurements of medial temporal lobe atrophy for prediction of Alzheimer's disease in subjects with mild cognitive impairment. Neurobiol. Aging.

[39] Yoshita M (2006). Extent and distribution of white matter hyperintensities in normal aging, MCI, and AD. Neurology.

[40] Smith EE (2008). Magnetic resonance imaging white matter hyperintensities and brain volume in the prediction of mild cognitive impairment and dementia. Arch. Neurol..

[41] Bennett DA (2005). Mild cognitive impairment is related to Alzheimer disease pathology and cerebral infarctions. Neurology.

[42] Nelson PT (2007). Clinicopathologic correlations in a large Alzheimer disease center autopsy cohort: neuritic plaques and neurofibrillary tangles "do count" when staging disease severity. J. Neuropathol. Exp. Neurol..

[43] Schneider JA (2009). The neuropathology of probable Alzheimer disease and mild cognitive impairment. Ann. Neurol..

[44] Gao YZ (2013). Regional cerebral blood flow and cerebrovascular reactivity in Alzheimer's disease and vascular dementia assessed by arterial spinlabeling magnetic resonance imaging. Curr. Neurovasc. Res..

[45] Binnewijzend MA (2014). Distinct perfusion patterns in Alzheimer's disease, frontotemporal dementia and dementia with Lewy bodies. Eur. Radiol..

[46] Alexopoulos P (2012). Perfusion abnormalities in mild cognitive impairment and mild dementia in Alzheimer's disease measured by pulsed arterial spin labeling MRI. Eur. Arch. Psychiatry Clin. Neurosci..

[47] Mak HK (2014). Combination of MRI hippocampal volumetry and arterial spin labeling MR perfusion at 3-Tesla improves the efficacy in discriminating Alzheimer's disease from cognitively normal elderly adults. J. Alzheimers Dis..

[48] Kilroy E (2014). Reliability of two-dimensional and three-dimensional pseudo-continuous arterial spin labeling perfusion MRI in elderly populations: comparison with 15O-water positron emission tomography. J. Magn. Reson. Imaging JMRI.

[49] Jack CR (2013). Tracking pathophysiological processes in Alzheimer's disease: an updated hypothetical model of dynamic biomarkers. Lancet Neurol..

[50] Jueptner M, Weiller C (1995). Review: does measurement of regional cerebral blood flow reflect synaptic activity? Implications for PET and fMRI. Neuroimage.

[51] Jack CR (2016). A/T/N: An unbiased descriptive classification scheme for Alzheimer disease biomarkers. Neurology.

[52] Fotuhi M, Do D, Jack C (2012). Modifiable factors that alter the size of the hippocampus with ageing. Nat. Rev. Neurol..

[53] Shea Y-F (2018). Impact of amyloid PET imaging in the memory clinic: a systematic review and meta-analysis. J. Alzheimer's Dis. JAD.

[54] Ikonomovic MD (2008). Post-mortem correlates of in vivo PiB-PET amyloid imaging in a typical case of Alzheimer's disease. Brain.

[55] Marcus C, Mena E, Subramaniam RM (2014). Brain PET in the diagnosis of Alzheimer's disease. Clin. Nucl. Med..

[56] Michaud TL (2017). The risk of incident mild cognitive impairment and progression to dementia considering mild cognitive impairment subtypes. Dement. Geriatr. Cognit. Disord. Extra.

[57] Busse A (2006). Mild cognitive impairment: long-term course of four clinical subtypes. Neurology.

[58] Ganguli M (2011). Outcomes of mild cognitive impairment by definition: a population study. Arch. Neurol..

[59] Ding D (2016). Progression and predictors of mild cognitive impairment in Chinese elderly: a prospective follow-up in the Shanghai Aging Study. Alzheimer's Dement. Diagn. Assess. Dis. Monit..

[60] Vemuri P (2009). MRI and CSF biomarkers in normal, MCI, and AD subjects: predicting future clinical change. Neurology.

[61] Donohue MC (2017). Association between elevated brain amyloid and subsequent cognitive decline among cognitively normal persons. JAMA.

[62] Vos SJ (2013). Preclinical Alzheimer's disease and its outcome: a longitudinal cohort study. Lancet Neurol..

[63] van Harten AC (2013). Cerebrospinal fluid Aβ42 is the best predictor of clinical progression in patients with subjective complaints. Alzheimers Dement.

[64] Jack CR (2008). 11 C PiB and structural MRI provide complementary information in imaging of Alzheimer's disease and amnestic mild cognitive impairment. Brain.

[65] Jhoo JH (2010). Discrimination of normal aging, MCI and AD with multimodal imaging measures on the medial temporal lobe. Psychiatry Res..

[66] Walhovd KB (2009). Multimodal imaging in mild cognitive impairment: Metabolism, morphometry and diffusion of the temporal–parietal memory network. Neuroimage.

